# REDUCED COGNITIVE DECLINE WITH THE APOE ε2/ε2 GENOTYPE IN THE LONG LIFE FAMILY STUDY AND NEW ENGLAND CENTENARIAN STUDY

**DOI:** 10.1093/geroni/igz038.2314

**Published:** 2019-11-08

**Authors:** Benjamin Sweigart, Benjamin Sweigart, Stacy L Andersen, Stephanie Cosentino, Nicole Schupf, Thomas T Perls, Paola Sebastiani

**Affiliations:** 1 Boston University, Department of Biostatistics, Boston, Massachusetts, United States; 2 Department of Biostatistics, Boston University School of Public Health, Boston, Massachusetts, United States; 3 Department of Medicine, Boston University School of Medicine, Boston, Massachusetts, United States; 4 Department of Neurology, Columbia University, New York, New York, United States; 5 Department of Epidemiology, Columbia University Mailman School of Public Health, New York, New York, United States

## Abstract

A growing body of evidence has suggested a protective effect on cognition of the ε2 allele of APOE. To determine if APOE ε2 is associated with protection against cognitive decline, we analyzed repeated measures of the Telephone Interview for Cognitive Status (TICS) from 2,933 Long Life Family Study subjects and 679 New England Centenarian Study subjects using a multivariable linear mixed effects model. The median age at first TICS administration was 73 (interquartile range [IQR] 64, 83). Subjects had a median of 3 TICS assessments (IQR 2, 4) and a median follow-up time of 5.0 years (IQR 2.9, 7.0). Carriers of the ε2/ε2 genotype had a significantly slower rate of decline in TICS score compared to the ε3/ε3 reference group (-0.05 points per annum for ε2/ε2 carriers compared with -0.15 points for ε3/ε3 carriers, p-value for difference 0.017). These results support a protective effect of the ε2 allele.

